# Editorial: Clinical Genome Sequencing: Bioinformatics Challenges and Key Considerations

**DOI:** 10.3389/fgene.2022.896032

**Published:** 2022-03-31

**Authors:** Shulan Tian, Zheng Jin Tu, Huihuang Yan, Eric W. Klee

**Affiliations:** ^1^ Division of Computational Biology, Department of Quantitative Health Sciences, Mayo Clinic, Rochester, MN, United States; ^2^ Molecular Pathology and Cytogenomics Division, Department of Laboratory Medicine, Cleveland Clinic, Cleveland, OH, United States

**Keywords:** bioinformatics, biomarker, next-generation sequencing, nomogram, RNA sequencing, microarray, whole-exome sequencing

Next generation sequencing (NGS) has been increasingly used to generate mutation, transcriptome and epigenomic profiles, as well demonstrated by The Cancer Genome Atlas (TCGA) (Tomczak et al., 2015) and the International Cancer Genome Consortium (ICGC) in major cancer types (Milius et al., 2014). It is evident that utilizing NGS-based omics data, individually or in combination, along with clinical metadata, can foster the development of robust biomarkers, such as tumor mutational burden, gene mutation and expression signature, and the classification of disease subtypes, thus benefiting patients in diagnosis, risk evaluation and potentially individualized therapy. In practice, however, prioritization on causal variants and genes still faces key challenges in data processing, harmonization, and clinical interpretation. Misinterpretation of genetic testing results remains a major bottleneck in cases of challenges (Farmer et al., 2021). This topic covers research articles that, as we described below, aimed to identify potentially functional variants and genes, or to build models for risk prediction.

Nomogram is a predictive model that is widely used to predict individual’s risk of recurrence, metastases and overall survival (Balachandran et al., 2015). To build a nomogram for early-stage hepatocellular carcinoma (HCC), Huang et al. downloaded transcriptome, mutation and clinical data for patients from a single cohort in TCGA and another four in ICGC. Cox regression analysis identified seven significant variables, including mutation status of *TP53*, *MACF1*, *EYS* and *DOCK2*, that were used to build the nomogram. The patients were then divided into low-versus high-risk group, with the former being associated with a better overall survival. Focused analysis of the cohort from TCGA revealed clear differences between the two risk groups in the abundance for seven of the 22 tumor-infiltrating hematopoietic cell subpopulations (Newman et al., 2015); also, the low-risk group had significantly lower Tumor Immune Dysfunction and Exclusion (TIDE) scores (Jiang et al., 2018), suggestive of a better immunotherapy response. This study demonstrated a risk stratification nomogram that is potentially linked to the infiltrating immune cell composition in HCC.

Starting with a public RNA-seq data of 117 Ewing sarcoma (ES) patients, Zhou et al. first calculated, for each sample, an immune enrichment score across each of the 28 infiltrating immune cell subpopulations (Jia et al., 2018), followed by unsupervised sample clustering. Two clusters with the highest and lowest overall score were retained. Of the differentially expressed genes (DEGs) between the two clusters, 862 formed a distinct immune-related module that showed the strongest negative correlation with immune score (estimated via the ESTIMATE package). About 10% (85 genes) were DEGs between normal skeletal muscle tissue and ES. They focused on *NPM1* (nucleophosmin 1) involved in DNA repair and cell proliferation, showing that its mRNA and protein expression levels were markedly higher in ES cell lines compared to mesenchymal stem cells. The higher mRNA expression correlated with lower immune score, TIDE score and *PD-L1* expression, as well as worse prognosis in ES. Importantly, NSC348884, a nucleophosmin inhibitor (Qi et al., 2008), can induce apoptosis in treated ES cells. This work recapitulates the previous finding that *NPM1*, a drug-targetable gene, is a prognostic biomarker in ES (Kikuta et al., 2009).

Through total RNA and miRNA sequencing, Wang et al. identified mRNAs, IncRNAs, and miRNAs differentially expressed between acute myeloid leukemia (AML) patients and healthy subjects. They used RAID, a comprehensive RNA-associated interaction database (Yi et al., 2017), to predict mRNAs and lncRNAs targeted by the differential miRNAs. The analysis revealed a potential network of the top 25 hub mRNAs with 15 miRNAs and 12 lncRNAs, including at least four mRNAs and two lncRNAs that are associated with overall survival. Notably, the expression of *CCL5* and lncRNA *UCA1*, known to play key roles in the proliferation of AML, correlated with the fraction of infiltrating immune and stromal cells (Yoshihara et al., 2013). The analysis also revealed a novel interaction between *UCA1* and *miR-16-5p*, expanding the known *UCA1*-miRNA crosstalk in AML (Sun et al., 2018). Together, this study supports *CCL5* and *UCA1* as potential diagnostic biomarkers in AML.

Biomarker discovery often relies on the integration of different datasets. In ulcerative colitis (UC), Chen et al. selected six microarray gene expression data from GEO, including 22–162 patients and 11–21 controls. After batch effect correction, 231–436 DEGs were identified from each dataset, with only 79 DEGs in common by a simple intersection approach. To effectively integrate the results, the authors applied the robust rank aggregation (RRA) method, which is robust to outliers and noises (Kolde et al., 2012), on the ranked DEG lists. Of the 208 RRA-identified DEGs, six hub genes were selected and confirmed to be upregulated in a UC mouse model. Indeed, these six genes are known to be associated with UC. Thus, to extract biological signatures shared across multiple datasets, one should consider robust meta-analysis approaches for high reproducibility.

Finally, Shestak et al. reported the genetic test of a 14-year-old female athlete, who was suspected to have long QT syndrome (LQTS). WES identified a rare mutation (c.647C > T, p. S216L, chr3:38655522-38655522) in the non-canonical exon 6 of *SCN5A*. *SCN5A* is a cardiac ion channel gene implicated in multiple cardiac diseases, with conclusive evidence for its causation in congenital LQTS (Adler et al., 2020). The clinical report, however, mistook this variant for the one previously reported in the canonical exon 6 (c.647C > T, p. S216L, chr3:38655290-38655290) (Marangoni et al., 2011), leading to misinterpretation. Subsequent Sanger sequencing confirmed a lack of mutation in canonical exon 6. Two more tests were ordered, and both identified the mutation only in the non-canonical exon 6. First, DNA was sequenced in a targeted panel of 11 genes including *SCN5A*, followed by Sanger sequencing validation. Second, Sanger sequencing revealed the mutation in the mother, but not in the father. The variant was classified as benign, suggesting negative result of the genetic testing. This study highlights the importance of variant validation. Obviously, the collaboration between clinicians and bioinformaticians is vital for genetic counseling. With the ongoing efforts, we are expecting the development of systems for accurately prioritizing causal variants and genes in accelerating biomarker discovery.
